# A brief multi-disciplinary review on antimicrobial resistance in medicine and its linkage to the global environmental microbiota

**DOI:** 10.3389/fmicb.2013.00096

**Published:** 2013-05-14

**Authors:** L. Cantas, Syed Q. A. Shah, L. M. Cavaco, C. M. Manaia, F. Walsh, M. Popowska, H. Garelick, H. Bürgmann, H. Sørum

**Affiliations:** ^1^Department of Food Safety and Infection Biology, Norwegian School of Veterinary ScienceOslo, Norway; ^2^Research Group for Microbial Genomics and Antimicrobial Resistance, National Food Institute, Technical University of DenmarkKgs Lyngby, Denmark; ^3^Centro de Biotecnologia e Química Fina, Escola Superior de Biotecnologia, Centro Regional do Porto da Universidade Católica Portuguesa, Rua Dr. António Bernardino AlmeidaPorto, Portugal; ^4^Bacteriology, Agroscope Changins-WädenswilWädenswil, Switzerland; ^5^Department of Applied Microbiology, Institute of Microbiology, University of WarsawWarsaw, Poland; ^6^Department of Natural Sciences, Middlesex UniversityLondon, UK; ^7^Department of Surface Waters - Research and Management, Eawag, Swiss Federal Institute for Aquatic Science and TechnologyKastanienbaum, Switzerland

**Keywords:** antimicrobial resistance, human and veterinary medicine, environment, soil, wastewater, resistance genes

## Abstract

The discovery and introduction of antimicrobial agents to clinical medicine was one of the greatest medical triumphs of the 20th century that revolutionized the treatment of bacterial infections. However, the gradual emergence of populations of antimicrobial-resistant pathogenic bacteria resulting from use, misuse, and abuse of antimicrobials has today become a major global health concern. Antimicrobial resistance (AMR) genes have been suggested to originate from environmental bacteria, as clinically relevant resistance genes have been detected on the chromosome of environmental bacteria. As only a few new antimicrobials have been developed in the last decade, the further evolution of resistance poses a serious threat to public health. Urgent measures are required not only to minimize the use of antimicrobials for prophylactic and therapeutic purposes but also to look for alternative strategies for the control of bacterial infections. This review examines the global picture of antimicrobial resistance, factors that favor its spread, strategies, and limitations for its control and the need for continuous training of all stake-holders i.e., medical, veterinary, public health, and other relevant professionals as well as human consumers, in the appropriate use of antimicrobial drugs.

## Background

Arguably one of the greatest examples of serendipity in science was the discovery of natural antimicrobials between Alexander Fleming and Ernest Duchesne. Although Fleming generally holds the reputation of the discovery of penicillin in 1928, a French medical student, Ernest Duchesne (1874–1912), originally discovered the antimicrobial properties of *Penicillium* earlier, in 1896. He observed Arab stable boys that kept their saddles in a dark and damp room to encourage mold to grow on them, which they said helped heal saddle sores. Curious, Duchesne prepared a suspension from the mold and injected it into diseased guinea pigs along with a lethal dose of virulent typhoid bacilli and still all animals remained healthy. His work, however, was ignored because of his young age and unknown status (Pouillard, 2002). In a way, with the success of the natural antibiotic penicillin and the synthetic antimicrobial sulfonamides in the first half of the 20th century, the modern antimicrobial revolution began. Since then, new natural antimicrobial compounds were discovered and many semi-synthetic and synthetic antimicrobial drugs were created to combat bacterial infections. Thus, antimicrobials have been extremely important corner stones of modern medicine since the last half of the previous century. Antimicrobial drugs have saved millions of people from life-threatening bacterial infections and eased patients' suffering. Today, the treatment of bacterial infections is once again becoming increasingly complicated because microorganisms are developing resistance to antimicrobial agents worldwide (Pouillard, 2002; Levy and Marshall, 2004; Alanis, 2005; Pallett and Hand, 2010).

A causal relationship has been demonstrated between the increased use of antimicrobials in both human and veterinary medicine, the greater movement of people, as well as domestic and wild animals, the increased industrialization and the increased prevalence of antimicrobial-resistant bacteria (Holmberg et al., 1987; Cheng et al., 2012). Antimicrobial resistance (AMR) was identified in pathogenic bacteria concurrently with the development of the first commercial antibiotic produced by microorganisms, penicillin (Abraham and Chain, 1940). Although the first concerns about drug-resistant bacteria appeared in hospitals, where most antimicrobials were being used (Levy, 1998), the importance of AMR was already recognized in 1969 both in humans and veterinary medicine as stated in the Swan Report (Swann, 1969). Further research showed that the origin and spread of AMR is, in fact, a very complex problem. Hence, there cannot be a single solution for minimizing AMR; rather a coordinated multi-disciplinary approach will be required to address this issue (Serrano, 2005; Smith et al., 2009). We must also recognize that wherever antimicrobials are used, AMR will inevitably follow.

**Figure 1:**
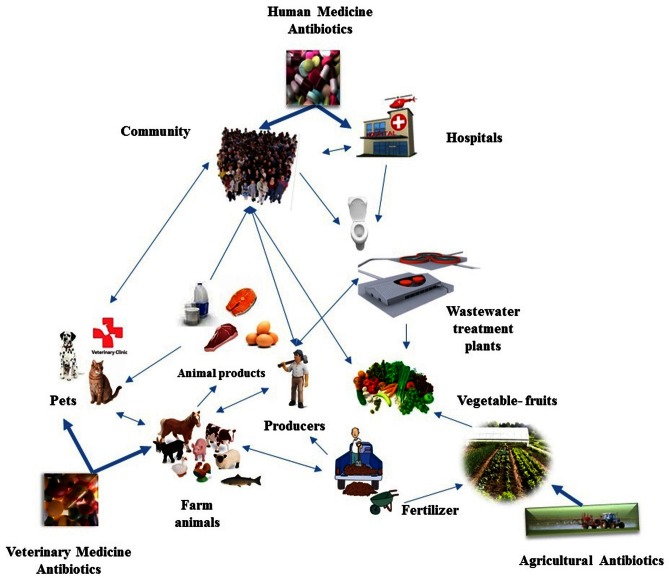
**Schematic representation of the complexity of the potential bacterial genetic web of communication between the various microbiotas that are impacted by the use of antibiotics in a wide context.** The reservoirs where antimicrobials are applied are also suggested as “hot spots” for horizontal gene transfer. The potentially most important genetic links between the microbiotas of the various reservoirs are showed by arrows. Thick arrows show major selective pressures for selection of antibiotic resistance genes, thin arrows show the significant directions of gene flow. Future research may document unique arrows that must be integrated in the web drawn.

The purpose of this review is to highlight the problem of resistance to antimicrobials with its consequences, including how the spread of AMR could be limited. We highlight how the numerous useful applications of antimicrobials led to AMR in different ecological locations (Figure 1), aiming to unify the many important aspects of this problem. Finally we advocate the need for teaching and continuous training of all stake-holders (i.e., medical, veterinary, public health, and other relevant professionals) as well as human consumers of antimicrobial drugs, in the appropriate use of antimicrobials.

## The human medicine and antimicrobial resistance

### Emergence of antimicrobial resistance and its cost

**Figure 2:**
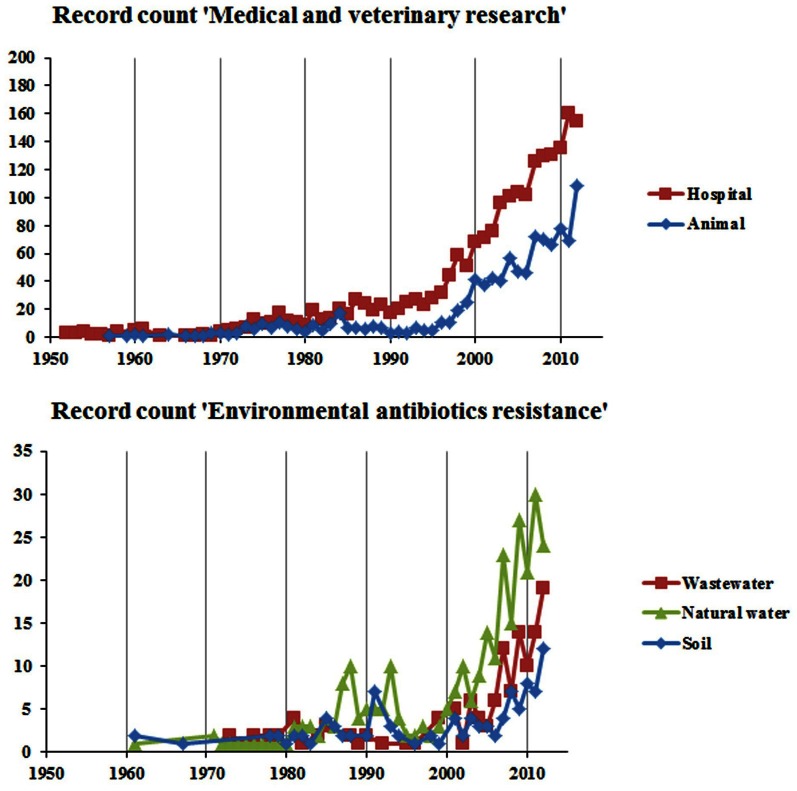
**The change in number of antimicrobial resistance related published research papers in different subdisciplines and covering different environments.** The data for the graphs were obtained by searching the ISI web of science for publications with titles matching the query terms (antibioti^*^ OR antimicro^*^) AND resistan^*^ AND the following specific terms: Hospital, (hospital^*^ OR patient^*^ OR clinic^*^); Animal, (animal^*^ OR veterinary^*^ OR livestock^*^ OR pig^*^ OR cow^*^ OR chicken^*^ OR poultry); Wastewater, (wastewate^*^ OR sewage); Natural water, (wate^*^ OR lake OR river OR ocean OR sea); Soil, (soil^*^ OR sediment^*^ OR rhizosphere^*^) (Source: http://apps.isiknowledge.com/). The search was performed on 06/03/2013.

In human medicine AMR is as old as the clinical usage of antimicrobial compounds. Antimicrobial-resistant pathogens have been observed soon after the introduction of new drugs in hospitals where antimicrobials are intensively used (Levy, 1998). Consequently, AMR in the context of human medicine has dominated the literature for a long time (Figure 2). Over the years, and continuing into the present, almost every known bacterial pathogen and numerous human commensals have developed resistance to one or more antimicrobials in clinical use (Table 1). Extended-spectrum β-lactamases (ESBL) are the ones most often encountered in the hospital (intensive care) setting. Methicillin-resistant *Staphylococcus aureus* (MRSA) and vancomycin-resistant enterococci (VRE) have also been found to have a significant nosocomial ecology (Otter and French, 2010). In addition, ESBL positive bacteria and MRSA infections are increasingly detected in the community. Furthermore, the increase in fluoroquinolone resistance due to target-site mutations and the worldwide emergence of plasmid-mediated quinolone resistance genes may represent a major challenge in future given the critical importance of this antimicrobial therapy (Cattoir et al., 2007; Strahilevitz et al., 2009). Carbapenems are the last line of defense against the non-Enterobacteriaceae pathogens, such as *Pseudomonas aeruginosa* and *Acinetobacter baumannii* (Brown et al., 1998). However, since the first description of the *bla*_OXA_ genes, there has been a worldwide increase in the dissemination of new resistance determinants conferring carbapenem resistance. For example, the *Klebsiella pneumoniae* carbapenemase (KPC) type enzymes, Verona integron-encoded metallo-β-lactamase (VIM), Imipenemase Metallo-β-lactamase (IMP) and New Delhi metallo-β-lactamase (NDM), and the OXA-48 type of enzymes have been isolated from a number of bacterial genera irrespective of their geographical distribution (Kumarasamy et al., 2010; Walsh et al., 2011). Carbapenemase resistance mechanisms are found among *Escherichia coli* and *Klebsiella* isolates in hospital settings, and to a lesser extent also in the community, thus healthy human carriers begin to be a concern (Nordmann et al., 2012). Furthermore, carbapenemase-producing organisms have also been isolated from farm animals (Fischer et al., 2012).

**Table 1:** **Antimicrobial resistance detection in some important pathogens soon after arrival of the “magic bullets” into the market**.

**Year**	**Bacteria**	**Drug resistance**	**Comments**	**References**
1948	*Staphylococcus aureus*	Penicillin	In British civilian hospitals soon after the introduction of penicillin	Barber and Rozwadowska-Dowzenko, 1948
1948	*Mycobacterium tuberculosis*	Streptomycin	In the community soon after the clinical usage of this antimicrobial	Crofton and Mitchison, 1948
1950's–1960's	*Escherichia coli, Shigella* spp., and *Salmonella enterica*	Multiple drugs		Watanabe, 1963; Olarte, 1983; Levy, 2001
1960's	VRE- *Enterococcus* spp. ESBL- *E. cloacae, K. pneumoniae, E. coli*, MRSA, Q^R^- Enterobacteriaceae MDR- *Acinetobacter baumannii, Pseudomonas aeruginosa*	Multiple drugs		Levy and Marshall, 2004; Nordmann et al., 2011

VRE, Vancomycin-Resistant Enterococcus; ESBL, Extended-spectrum β-lactamase; MRSA, methicillin/oxacillin-resistant Staphylococcus aureus; Q^R^, Quinolone resistant; MDR, Multi-drug resistant.

### Human mobility—the direct and indirect impact on human pathogens

The increasing cross-border and cross-continental movements of people has a major impact on the spread of multi-resistant bacteria (Linton et al., 1972; Arya and Agarwal, 2011; Cheng et al., 2012). The emergence and global spread of the international clone 1 of penicillin-resistant *Streptococcus pneumoniae* (Klugman, 2002) and the recently occurring New Delhi Metallo-β-lactamase (*bla*_NDM_-1) producing *Enterobacteriaceae*, which inactivates all β-lactam antimicrobials, including carbapenems, are good examples. The *bla*_NDM_-1 appears to have originated in the Indian subcontinent and subsequently could be found in North America, the United Kingdom, and other European countries by the movement of people (Arya and Agarwal, 2011; Walsh et al., 2011).

The AMR problem remains a growing public health concern because infections caused by resistant bacteria are increasingly difficult and expensive to treat. The consequences of this problem are: longer hospital stay, longer time off work, reduced quality of life, greater likelihood of death due to inadequate or delayed treatment, increases in private insurance coverage and an additional costs for hospitals when hospital-acquired infections occur in addition to the increased overall healthcare expenditure (Roberts et al., 2009; Filice et al., 2010; Korczak and Schöffmann, 2010; Wilke, 2010). Thus, in order to calculate the full economic burden of AMR we have to consider the burden of not having antimicrobial treatment options at all, which in the extreme case would probably cause a breakdown of the entire modern medical system (Alanis, 2005; Falagas and Bliziotis, 2007; Pratt, 2010). In short, everyone will be at risk when antimicrobials become ineffective and the threat is greatest for young children, the elderly, and immune-compromised individuals, such as cancer patients undergoing chemotherapy and organ transplant patients (Tablan et al., 2004).

## The veterinary medicine and agriculture sector

### Consumption and regulation of antimicrobial use in animals

The antimicrobials, used in veterinary medicine were introduced soon after they became available for the treatment of human diseases from the mid-1940's (Gustafson and Bowen, 1997; McEwen, 2006). Even though some drugs are exclusively designed for veterinary use, most belong to the same antimicrobial classes as those used in human medicine with identical or very similar structures (Swann, 1969; Heuer et al., 2009).

Annually, large quantities of drugs are administered to animals in the agricultural sector worldwide to secure a sufficient amount of food (meat, eggs, and dairy products) to feed a rapidly growing world human population (Vazquez-Moreno et al., 1990; Roura et al., 1992; Rassow and Schaper, 1996). As data collection on antimicrobial use in animals was not harmonized to provide reliable and comparable information, and following a request from the European Commission the European Medicines Agency (EMA), the European Surveillance of Antimicrobial Consumption (ESVAC) programme has been created. The ESVAC programme is responsible for collecting, analyzing, and reporting sales data from European countries and developing an organized approach for the collection and reporting of data on antimicrobial use for animals including annual reporting from EU member states (www.ema.europa.eu/ema/index.jsp?curl=pages/regulation/document_listing/document_listing_000302.jsp). During 2007, in 10 European countries the sale of antimicrobial drugs for therapeutic use as veterinary medicine varied from 18 to 188 mg/kg biomass (Grave et al., 2010).

The administration of antimicrobials to food producing animals can have other purposes than treatment, such as: growth promotion (although now totally banned in Europe and quinolones for the poultry industry are banned in the USA), prophylaxis, and -metaphylaxis (Anthony et al., 2001; Anderson et al., 2003; Casewell et al., 2003; Cabello, 2006). Approximately 70% of all the antimicrobials administered in animal farming are used for non-therapeutic purposes (Roe and Pillai, 2003).

In the European Union (EU) the use of avoparcin was banned in 1997. Furthermore spiramycin, tylosin, and virginiamycin for growth promotion were banned from use in 1998. All other growth promoters in the feed of food-producing animals were banned in the EU—countries from January 1, 2006 (http://europa.eu). Data from Denmark showed that animals could be produced at a large scale without the use of growth promoters, without adversely affecting the production (Aarestrup et al., 2001; Aarestrup, 2005; Hammerum et al., 2007). In the United States of America, politicians are discussing the introduction of a similar ban on the use of antimicrobials in animal husbandry for growth promotion (http://www.govtrack.us/congress/bills/109/s742). Despite these bans, in some parts of the world, medically important antibiotics are still routinely fed to livestock prophylactically to increase profits and to ward-off potential bacterial infections in the stressed and crowded livestock and aquaculture environments (Cabello, 2006; Smith et al., 2009; Ndi and Barton, 2012). Because stress lowers the function of the immune system in animals, antimicrobials are seen as especially useful in intensive confinements of animals (Halverson, 2000). The non-therapeutic use of antimicrobials involves low-level exposure through feed over long periods—an optimal way in which to enrich resistant bacterial populations (Sharma et al., 2008; Kohanski et al., 2010; Alexander et al., 2011; Gullberg et al., 2011).

Various monitoring programs around the world have started monitoring AMR and a range of research activities and interventions have shown that antimicrobial usage has a large effect upon selection of AMR in animal production. A rapid and parallel decrease in resistant *Enterococcus faecium* from pig and poultry has been reported in Denmark after the ban of growth promoters in livestock (Aarestrup et al., 2001). The Norwegian aquaculture industry has produced over one million tons of farmed fish (http://www.ssb.no/fiskeoppdrett_en/) by using only 649 kg of antimicrobials in 2011 (NORM/NORM-VET, 2011). It is evident from the Danish integrated AMR monitoring and research program (DANMAP) and NORM/NORM-VET Report (NORM/NORM-VET, 2011) that reduction of antimicrobial usage with strict policies may still be the safest way to control the development and spread of AMR in this sector in the future.

### Antimicrobial-resistant bacteria in companion animals and animal husbandry

The use of antimicrobials in animal husbandry has for many years actively selected for bacteria which possess genes capable of conferring AMR (Bastianello et al., 1995; Sundin et al., 1995). Consequently, this aspect has also seen much attention in the literature (Figure 2). Despite large differences in methodology, the results of most relevant scientific studies demonstrate that not long after the introduction of antimicrobials in veterinary practice, resistance in pathogenic bacteria, and/or the fecal flora was observed (Caprioli et al., 2000; Jean-Louis et al., 2000). In particular an increased emergence of pathogenic bacteria resistant to antimicrobials has occurred in members of the genera *Salmonella, Campylobacter, Listeria, Staphylococcus, Enterococcus*, and *Escherichia coli.* Some resistant strains of these genera are propagated primarily among animals but can subsequently infect people as zoonotic agents (Levy, 1984; Corpet, 1988; Marshall et al., 1990; Giguêre et al., 2007).

In veterinary medicine the use of antimicrobials in companion animals such as pets and horses is restricted to therapeutic purposes only. Companion animals are increasingly treated as family members, in the context of applying advanced antimicrobial treatments to their infectious diseases. For instance, skin infections caused by staphylococci in dogs with or without underlying allergic reactions result in an increasing use of semi-synthetic penicillins because of the ineffectiveness of penicillin against penicillinase producing *Staphylococcus pseudintermedius* (Yoon et al., 2010). Moreover, emerging methicillin-resistant *Staphylococcus pseudintermedius* (MRSP), methicillin-resistant *Staphylococcus aureus* (MRSA), and ESBL producing *E. coli* displaying multidrug resistance has led to increased concern related to AMR in companion animal practice (Bannoehr et al., 2007; Wieler et al., 2011). Increased antimicrobial resistance development and spread in companion animals due to irrational antimicrobial usage, especially overprescribed broad spectrum antimicrobials without precise diagnostics, inevitably cause (1) animal health problem (increased mortality and morbidity), (2) economical problem to the owner (more visits-therapies and prolonged hospitalization), (3) economical problem to the veterinarian (possible loss of customers and high costs for hospital decontamination), and (4) human health problems (risks of zoonotic transmission). Because of this threat small animal veterinarians should prescribe broad spectrum antimicrobials after culturing and educate pet owners to handle infected-antimicrobial treated animals with precaution.

However, emergences of resistance toward antimicrobials which are critically important for human therapy are the most worrisome. These include the recent emergence of ESBL producing and carbapenemase positive *Enterobacteriaceae* bacteria in animal production (Horton et al., 2011), the emergence of farm associated MRSA ST398 (the main pig associated clone) (Cuny et al., 2010; Kluytmans, 2010; Weese, 2010) and of plasmid-mediated quinolone resistance in animal isolates and food products (Poirel et al., 2005; Nordmann et al., 2011). Unfortunately, there are several examples in the literature that show that these are already widespread in Europe and other parts of the world and have a large impact on human health (Angulo et al., 2004; Heuer et al., 2009; Forsberg et al., 2012).

Aquaculture (fish, shellfish, and shrimp farming industries) has developed rapidly in the last decade and has become an important food source (FAO, 2010). Fish pathogenic bacteria often produce devastating infections in fish farms where dense populations of fish are intensively reared. Although modern fish farming relies increasingly on vaccination and improved management to avoid infections (Markestad and Grave, 1997; Midtlyng et al., 2011), still many bacterial infections in fish are regularly treated with antimicrobials in medicated feed or by bath immersion. The most widely used drugs are fluoroquinolones, florfenicol, oxytetracyclines amoxicillin and sulfonamides (Cabello, 2006; Gräslund et al., 2003; Holmström et al., 2003; Primavera, 2006; Soonthornchaikul and Garelick, 2009). By now, most of the fish pathogenic bacteria from fish farms with a history of infections have developed AMR (Colquhoun et al., 2007; Lie, 2008; Sørum, 2008; Farmed Fish Health Report, 2010; Shah et al., 2012a). Furthermore, in some areas of the world, particularly in South-East Asia, integrated farming is a common practice where organic wastes from poultry and livestock are widely used in manuring the fish farms (Hoa et al., 2011; Shah et al., 2012b). It has been reported that antimicrobial residues present in the poultry and livestock waste has provided sufficient selection pressure for the selection of AMR genes, increasing the complexity of transmission of bacteria, and resistances between the livestock and aquatic environment (Petersen et al., 2002; Agersø and Petersen, 2007; Hoa et al., 2011; Shah et al., 2012b).

AMR has been detected in different aquatic environments and some resistance determinants have been found to originate from aquatic bacteria. A good example is the recently emerging plasmid-mediated quinolone resistance determinants from the *qnr* family (Ash et al., 2002; Picao et al., 2008) and CTX-M from aquatic *Kluyvera* spp. (Decousser et al., 2001; Rodriguez et al., 2004; Ma et al., 2012). In addition, epidemiological and molecular data indicate that some fish pathogens such as *Aeromonas* are able to transmit and share AMR determinants with bacteria isolated from humans such as *E. coli* (Rhodes et al., 2000; Sørum, 2006). Similarly, the fish pathogen *Yersinia ruckeri* have been reported to share AMR plasmid and AMR genes with the bacterium causing human plague (Welch et al., 2007).

### Antimicrobial use in plant agriculture

Streptomycin and oxytetracycline are routinely used for the prophylaxis of fire blight disease (causative agent *Erwinia amylovora*) in apple and pear orchards. Streptomycin use is strictly controlled within the EU and is only authorized for use on a yearly basis. However, streptomycin use in plant agriculture in the USA has been replaced by oxytetracycline, due to streptomycin resistance development among *E. amylovora* in the apple orchards. Oxolinic acid had been reported to be used in Israel against fire blight and against rice blight in Japan (Shtienberg et al., 2001). Gentamicin is used in Mexico and Central America to control Fire Blight and various diseases of vegetable crops (Stockwell and Duffy, 2012). However, the role of antimicrobial use on plants is, knowing the AMR crisis in human medicine, the subject of debate (McManus et al., 2002).

### Dissemination of antimicrobial-resistant bacteria through food and food production

Resistant bacteria can be transferred from animals and plants to humans in many different ways, which can be categorized into three major modes of transmission: (1) through the food chain to people (Roe and Pillai, 2003; Soonthornchaikul and Garelick, 2009), (2) through direct or indirect contact with livestock industry or animal health workers (Levy et al., 1976), (3) through environments which are contaminated with manure in agriculture (http://ec.europa.eu/environment/integration/research/newsalert/pdf/279na4.pdf) and aquaculture (Petersen et al., 2002; Shah et al., 2012b). The environment contains a great variety of bacteria creating an immense pool of AMR genes that are available for transfer to bacteria that cause human disease (Riesenfeld et al., 2004b; D'Costa et al., 2006). The realization of these links sparked the recent interest in the role and dynamics of environmental AMR (Figure 2).

In addition, other sources are available. For instance, wild animals may also be carriers of antimicrobial-resistant bacteria (Literak et al., 2011). These animals may have close contact to human or farming areas and/or waste and become colonized with resistant strains (Literak et al., 2011; Nkogwe et al., 2011). Interestingly, animals in remote areas have been found to harbor-resistant bacteria (Zhang et al., 2009; Glad et al., 2010; Lang et al., 2010).

## Antimicrobial resistance in the environment

### Microbial communities in soil and antimicrobial resistance

Research data shows that in diverse soils from various regions of the world, there is a wide dispersion of AMR. One explanation for this phenomenon is the existence of antimicrobial producing bacteria in soil. The Actinomycetes, which are common soil bacteria (*Streptomyces, Micromonospora, Saccharopolyspora* genus), synthesize over half of all known most clinically relevant antimicrobials e.g., tetracycline, gentamicin, erythromycin, streptomycin, vancomycin, and amphotericin. Bacteria of the genus *Bacillus* also produce antibiotics, e.g., *Bacillus brevis* which producing gramicidin (Baltz, 2007). These antimicrobials now also reach the environment from human and animal therapeutics, through manure, sewage, agriculture, etc. Many retrospective and prospective studies have been performed to assess the emergence and selection of AMR in environmental bacteria. The environment is eventually the largest and most ancient reservoir of potential AMR, constituting the environmental “resistome” (Aminov and Mackie, 2007; Allen et al., 2010; D'Costa et al., 2011). Under such powerful selection pressure, it is not surprising that the soil resistome is so diverse (Knapp et al., 2010). The best example illustrating this is the tetracycline resistome. Tetracyclines are an important class of antimicrobials with desirable broad-spectrum activity against numerous pathogens and the widespread emergence of resistance has had a massive impact on these drugs (Thaker et al., 2010). Opportunistic pathogens ubiquitous in the soil for example, *Pseudomonas aeruginosa, Acinetobacter* spp.*, Burkholderia* spp., and *Stenotrophomonas* spp. can combine intrinsic resistance to several antimicrobials with a remarkable capacity to acquire new resistance genes (Popowska et al., 2010). Still, little is known about the diversity, distribution, and origins of resistance genes, particularly among the as yet non-cultivable environmental bacteria. In uncultured soil bacteria, identified resistance mechanisms comprise efflux of tetracycline and inactivation of aminoglycoside antimicrobials by phosphorylation and acetylation (Popowska et al., 2012). In addition, bacteria resistant to macrolides including the new drug telithromycin have been reported from soil (Riesenfeld et al., 2004a). In a study by (D'Costa et al., 2006), 480 strains of *Streptomyces* from soil were screened against 21 antimicrobials. Most strains were found to be multi-drug resistant to seven or eight antimicrobials on average, with two strains being resistant to 15 of the 21 drugs. It was also reported that soil is a reservoir for β-lactamases and these genes, if transferred to pathogens, can then impact human health (Allen et al., 2010). It is supposed that the presence of antibiotics in the environment has promoted the acquisition or independent evolution of highly specific resistance elements. These determinants are located mainly on mobile genetic elements such as plasmids and conjugative transposons, which ensure their spread by horizontal gene transfer. Conjugative, broad-host-range plasmids play a key role in this process (Martinez, 2009; Stokes and Gillings, 2011). Numerous studies have demonstrated that the prevalence of such resistance plasmids in soil is very high (Götz et al., 1996). Among the plasmids conferring resistance to antimicrobials, representatives of the incompatibility groups P, Q, N, and W have been identified. An example of this type of mobile genetic elements may be the IncP-1 plasmids (Popowska and Krawczyk-Balska, 2013). Results from the scientific literature show that plasmids carrying resistance genes have been identified in pathogenic bacteria of the genus *Escherichia, Salmonella, Shigella, Klebsiella, Aeromonas*, and *Pseudomonas*, the genera that can be found in soil and water (Stokes and Gillings, 2011). These plasmids carry determinants for resistance to at least one heavy metal (Ni, Cd, Co, Cu, Hg, Pb, Zn) and antimicrobials of different groups, i.e., tetracyclines, quinolones, aminoglycosides, sulfonamides, β-lactams, and chemotherapeutics (Sen et al., 2011; Seiler and Berendonk, 2012). Overall these data indicate that soil bacteria constitute a reservoir of resistance determinants that can be mobilized into the microbial community including pathogenic bacteria. Recent studies also indicate a different mechanism of AMR in soil-derived actinomycetes, by engendering mutations in genes encoding the transcriptional and translational apparatus that lead to alterations in global metabolism. This vertically selected AMR includes increased production of secondary metabolites (Derewacz et al., 2013). Very recently evidence for recent exchange of AMR genes between environmental bacteria and clinical pathogens was presented using a high-throughput functional metagenomic approach (Forsberg et al., 2012). In this study it was shown that multidrug-resistant soil bacteria contain resistance gene cassettes against five classes of antimicrobials (β-lactams, aminoglycosides, amphenicols, sulfonamides, and tetracyclines) with high nucleotide identity to genes from diverse human pathogens. Therefore, it is important to study this reservoir, which may contribute to the detection of new clinically relevant AMR-mechanisms and/or the multidrug-resistant pathogens that should be avoided from entering medically important bacteria (Torres-Cortés et al., 2011).

### Antimicrobial resistance in aquatic environments

Water is one of the most important habitats for bacteria, holding complex microbial communities. Not surprisingly, water also contains AMR bacteria. From natural fresh water systems to drinking water, or from sewage to human-engineered water infrastructures, AMR, either intrinsic or acquired, have been reported in aquatic environments worldwide (e.g., Goñi-Urriza et al., 2000; Volkmann et al., 2004; Schwartz et al., 2006; Ferreira da Silva et al., 2007; Böckelmann et al., 2009; Vaz-Moreira et al., 2011; Falcone-Dias et al., 2012). In this respect, given their characteristics, wastewater habitats are particularly important.

### Wastewater habitats as reservoirs of antimicrobial resistance

Among the aquatic environments, wastewater habitats represent the most important reservoir of AMR bacteria and genes. This type of water contains human and animal excretions with abundant doses of commensal and pathogenic antimicrobial-resistant bacteria (Yang et al., 2011; Ye and Zhang, 2011, 2013; Novo et al., 2013). Since antimicrobials are not fully degraded in the human and animal body, antimicrobial compounds, their metabolites and transformation products are abundant in urban sewage treatment plants (Segura et al., 2009; Michael et al., 2013). Although proportion of the antimicrobial compounds are transformed and degraded in the environment, the occurrence of these micropollutants is reported worldwide, with antimicrobials of all classes being detected in wastewater habitats in concentrations ranging from ng^−1^ to mgL^−1^ (Michael et al., 2013). Simultaneously, urban sewage and wastewater contain AMR bacteria and other pollutants, such as pharmaceutical and personal hygiene products and heavy metals, whose effects on AMR selection are still not very clear (Graham et al., 2011; Oberlé et al., 2012; Patra et al., 2012; Novo et al., 2013). Often, wastewater treatment is not enough to eliminate the antimicrobial residues entering the system (Michael et al., 2013). The consequence is that such micropollutants, exerting selective pressures, may facilitate the selection of AMR bacteria or the acquisition of resistance genes by horizontal gene transfer (Martinez, 2009). Indeed, the relevance of wastewater habitats to the dissemination of AMR among human pathogens as well as commensal and environmental bacteria is increasingly emphasized (Baquero et al., 2008; Marshall and Levy, 2011; Czekalski et al., 2012; Rizzo et al., 2013). Wastewater treatment plants reduce the load of AMR bacteria, but treated water still carries elevated levels of AMR bacteria, and may select for strains with high levels of multidrug-resistance (Czekalski et al., 2012). Resistance gene abundance in a stream system could be linked to the input of (treated) wastewater and animal husbandry, demonstrating landscape-scale pollution of natural aquatic systems with AMR (Pruden et al., 2012). The currently available literature demonstrates that most of the AMR genetic elements found in clinical isolates are also detected in wastewater habitats, even shortly after they have been reported in hospitals (Szczepanowski et al., 2009; Rizzo et al., 2013). The occurrence of the same AMR genetic elements in different habitats demonstrates the uniqueness of the resistome, mainly due to rapid dissemination processes, demonstrating the urgent needs for an integrated approach.

Ubiquitous bacteria that can live in the environment and are also able to colonize humans are particularly relevant to the spread of AMR in the environment and the implications to human health. Indeed, numerous studies have reported the occurrence of AMR in ubiquitous bacteria isolated from wastewater habitats, which are also recognized as opportunistic pathogens, mainly nosocomial agents. AMR bacteria of clinical relevance which may be found in the environment comprise, among others, members of the genera *Acinetobacter, Enterococcus, Escherichia, Klebsiella, Pseudomonas*, and *Shigella* (Blanch et al., 2003; Reinthaler et al., 2003; Ferreira da Silva et al., 2006, 2007; Watkinson et al., 2007; Novo and Manaia, 2010; Czekalski et al., 2012). In addition, non-cultivable bacteria may also be important either for AMR spread or selection. Indeed, over the last years, the use of culture independent approaches brought additional insights into the abundance and diversity of resistance genes in wastewaters and into the effects of antimicrobials on the bacterial communities (Volkmann et al., 2004; Czekalski et al., 2012; Oberlé et al., 2012; Novo et al., 2013). In particular, several studies presented evidence that in wastewater habitats there is a high potential for horizontal gene transfer, mediated by plasmids and facilitated by integrons (Tennstedt et al., 2003; Szczepanowski et al., 2008; Moura et al., 2010; Zhang et al., 2011). Despite the importance of wastewater as a reservoir for AMR genes, and the relevance of wastewater treatment to control resistance spread, to date the number of studies that have been published remains relatively low (Figure 2).

Nevertheless, over the last decades the knowledge in this area has increased considerably and the importance of wastewater treatment systems for the spread of AMR is unequivocally demonstrated. Therefore, it is now possible to address some specific questions which we expect to be a focus of the research in this area in the coming years. Examples of these issues are (1) the identification of the conditions that may enhance or mitigate the occurrence of horizontal gene transfer and selection of AMR (which pollutants, which concentrations, temperature, pH, hydraulic residence time of wastewater treatment, etc.); (2) the classification and quantification of risk, e.g., the likelihood that an AMR bacterium or gene from wastewater habitats reach humans and causes issues for human health; (3) the improvement of wastewater treatment processes in order to minimize the loads of antimicrobial-resistant bacteria and genes in the final effluent (Dodd, 2012).

### The antimicrobial resistance gene pool

AMR genes can be differentiated depending on the genetic event that is required for acquiring an AMR phenotype. These include genes that are acquired by horizontal gene transfer and genes that are present in the bacterial genome and that can encode AMR following gene mutations or activation (Olliver et al., 2005).

AMR features evolve as a consequence of permanent exchange of and ever new recombinations of genes, genetic platforms, and genetic vectors. Many of these genes are not primarily resistance genes, but belong to the hidden resistome, the set of genes able to be converted into AMR genes (Baquero et al., 2009). As evidenced by our discussion above, microbial organisms harboring these genes are present naturally in all kinds of environments, but also released into water and soil from organisms, including humans, where they evolve or increase in abundance under direct selection from exposure to antimicrobials. At the same time, antimicrobials (often at low concentrations), disinfectants, and heavy metals are disseminated into the water as well, and may act as selective factors fostering the evolution of new AMR features (Cantas et al., 2012a,b,c; Cantas et al., unpublished). The rate of degradation of antimicrobials in the environment varies and is dependent on a range of environmental conditions, for example: temperature, available oxygen, pH, presence of alternative sources of organic and inorganic discharges as described in Table 2.

**Table 2:** **Degradation rates of various antimicrobials in soil**.

**Class of antimicrobial**	**Degradation [%]**	**Time [d]**	**References**
Macrolides	0–50	5–30	Thiele-Bruhn, 2003^*^
Sulfonamides	0–50	22–64	Thiele-Bruhn, 2003
Fluoroquinolones	0–30	56–80	Hektoen et al., 1995; Thiele-Bruhn, 2003
Tetracycline	0–50	10–180	Björklund et al., 1991; Thiele-Bruhn, 2003
Aminoglycosides	0	30	Thiele-Bruhn, 2003
β-lactams	0–50	30	Thiele-Bruhn, 2003
Imidasoles	50	14–75	Thiele-Bruhn, 2003
Polypeptides	12–90	2–173	Thiele-Bruhn, 2003

* This reference does not include the modern macrolides with very long elimination half-lives. For instance, Tulathromycine has an half live (so 50% degraded, not nearly 100%) in soil of 99 days (Pfizer, personal communication 2013).

### How to slow down the spread and evolution of AMR?

In this review we have emphasized that the problem of AMR evolution and dissemination is multifaceted and involves clinical, agricultural, technical, and environmental systems. Similarly strategies to deal with the impending AMR crisis have to take this complexity into account.

The overuse of antimicrobials needs to be limited or reduced in human medicine, veterinary medicine, agriculture, and aquaculture. Ideally, the use of antimicrobials in agriculture should be eliminated. Intensive programs to educate both patients and physicians in reducing antimicrobial overuse should be implemented. Following the analysis more than 500 scientific articles, it has been suggested that the elimination of non-therapeutic use of antimicrobials in food animals, will lower the burden of AMR in the environment, with consequent benefits to human and animal health (FAAIR Scientific, 2002; Swartz, 2002).

Better management techniques and strict legislation in the use of antimicrobials for therapeutic use in humans and in animals will reduce the risk of development of AMR (Cunha, 2002; Defoirdt et al., 2011; Midtlyng et al., 2011). For example, the prevention of nosocomial transmission of multi-drug resistant bacteria is possible with active routine surveillance programs that can identify colonized patients. Numerous studies have demonstrated that such a “search and containment” approach and/or a “search and destroy” approach in which an attempt is made to eliminate carriage of the organism can reduce the incidence of hospital-acquired infections and be cost-saving (Muto et al., 2003).

New management techniques in the animal husbandry, such as organic farming, need to be thoroughly investigated to ensure that these are viable alternatives that help to reduce the potential for selection of AMR bacteria. Samples from organically farmed poultry showed a significantly lower level of AMR in intestinal bacteria such as *E. coli* and *Campylobacter* (Soonthornchaikul et al., 2006). However from organically farmed cattle no significant differences were obtained in microbiological contamination. *E. coli* and *S. aureus* isolates were found to have significantly lower rates of AMR in organically raised cattle (Sato et al., 2005). More studies are needed (1) to determine the reasons of antimicrobial usage in the farms by veterinarians, (2) to compare and update the recommended treatment protocols for veterinarians throughout different countries, (3) to evaluate the impact of other factors other than AMR development in bacteria: e.g., immune response-stress has been indicated to correlate with resistance genetic element shuffling among gut microbiota in different animal models, such as: atlantic salmon, zebrafish, neonatal piglets, and cats (Cantas et al., 2011, 2012a,b,c, 2013). Animal welfare parameters under intensive production such as stress should be investigated in future studies with regards to control of resistance development in animal husbandry.

Vaccination and improved hygienic measures are among the important cornerstones in controlling infectious diseases and consequently aid in reducing AMR (Potter et al., 2008). The Norwegian aquaculture may serve as a good example by reduction in the use of antimicrobials from around 50 tons in the late 1980's to less than 1000 kg per annum after introduction of effective vaccines against devastating fish diseases like furunculosis and vibriosis (Midtlyng et al., 2011; NORM/NORM-VET, 2011).

The use of pre- and probiotics to improve the health and performance of livestock might be a good alternative to growth promoters. This is an important biological control aiming to reduce outbreaks of infectious diseases and which in turn would minimize the use of antimicrobials in livestock and aquaculture for therapeutic purposes (Verschuere et al., 2000; Callaway et al., 2008).

The issue of dissemination and possible long-term enrichment of AMR and AMR genes in the environment (Knapp et al., 2008) needs to be studied further, with specific regards to the actual risks associated with it. However, taking action is already possible today. For example, several treatment methods for waste and wastewater disinfection and removal of micropollutants, including antimicrobials, are available. These include various chemical disinfections, UV treatment, and membrane filtration. Disinfection and DNA degradation of community based and hospital wastewater may be effective means to reduce AMR release, although more research is required to fully assess the inactivation of resistance genes (i.e., DNA released from lysed cells that may be available for horizontal gene transfer) by these measures (Dodd, 2012). The combined removal of pollutants that are potential selective agents, disinfection, and deactivation of the genetic material, may be a useful strategy to reduce the pollution of environments with resistance factors.

## Conclusions

Inevitably, AMR in medicine has become common place. Bacteria have evolved multiple mechanisms for the efficient evolution and spread of AMR. Meanwhile the new developments of quick and adequate molecular diagnostic techniques for the identification and epidemiological surveillance of genetic determinants of AMR in different hosts and in the environment will enhance the number of control options. We have outlined above a number of potential measures that are enabled by our improved ability to track AMR. However, there seems to be a clear need for action and policy changes. This includes drug licensing, financial incentives, penalties, and ban or restriction on use of certain drugs. Similarly, the prescriber behavior needs to be altered. Animal health and hygiene needs to be improved. In addition, the implementation of microbiological criteria for the detection of certain types of resistant pathogens would be important to control the trade of both food animals and food products. The problem of AMR in human medicine will not be solved if nothing is done to limit the constant influx of resistance genes into the human microbiota via the food chain or contact with the environment. Introduction of antimicrobial compounds into the aquatic environment via medical therapy, agriculture, animal husbandry and companion animals has resulted in selective pressures on resident environmental bacteria. Development of AMR in environmental bacteria has a great impact and may help in explaining how human and animal pathogens acquire resistance features. Besides the role of clinical microbiology laboratories with rapid and accurate detection of a diverse number of pathogens and its drug resistance profiles, robust routine surveillances in an epidemiological frame-work covering the whole livestock “food chain” and the environment need to be taken into consideration. Due to this complexity the control of AMR has to include numerous actions at diverse levels. Future research should focus on finding unknown routes of transfer of AMR between microbiotas of relevance to the food chain and to all microbiotas of importance for bacterial pathogens when they acquire antibiotic resistance genes laterally. Ultimately, even a successful integrative approach on all aspects, can probably only help to slow down the spread of AMR, not prevent it. The development of new generations of antimicrobial should therefore receive equal attention. This is summarized and emphasized in a 12-point action plan against the rising threats of AMR implemented by the European Commission which includes actions in the field of human medicine, animal husbandry, veterinary medicine, authorization requirements for commercialization of human and veterinary drugs and other products, on research, on scientific opinions, and undertaking also actions on the international level in collaboration with the WHO and Codex (http://ec.europa.eu/dgs/health_consumer/does/communication_amr_2011_748_en.pdf, Bush et al., 2011).

The problem of AMR is widespread all over the world, therefore it is not eradicable, but can be managed. Concerted efforts between medical doctors, dentists, veterinarians, scientists, funders, industry, regulators, and multi-disciplinary approaches are needed to track resistance. Furthermore, global monitoring of the antimicrobial drug consumption in human and veterinary medicine and AMR, is an essential part of an overall strategy to inform, educate and get commitment of all parties, including farmers and patients (American Academy of Microbiology, 2009). All these are important measures for the efficient future use of antimicrobials in medicine. All members of society should be conscious of their role and take on responsibility for maintaining the effectiveness of current and future antimicrobials. We believe that future interventions can be successful in minimizing this problem.

## Author contributions

L. Cantas defined the review theme, established the interdisciplinary coordination and the collaborations, designed the manuscript, contributed to the data collection, data analysis, and drafting and writing of the manuscript. Syed Q. A. Shah, L. M. Cavaco, C. M. Manaia, F. Walsh, M. Popowska: drafting, writing, and editing the manuscript. H. Garelick and H. Bürgmann: contributed to data analysis, drafting, and writing the manuscript. H. Sørum: contributed to manuscript design, data collection, data analysis, drafting, and writing the manuscript. All authors have contributed to, seen and approved the manuscript.

## Acknowledgments and funding

**Figure:**
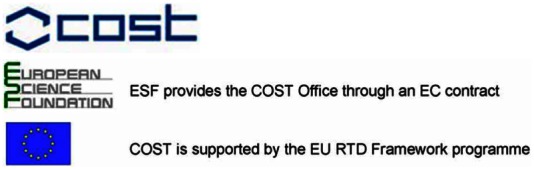


All authors acknowledge DARE TD0803 (Detecting Antimicrobial Resistance in Europe) COST Action. (http://www.cost-dare.eu/). This publication is supported by COST. Neither the COST Office nor any person acting on its behalf is responsible for the use which might be made of the information contained in this publication. The COST Office is not responsible for the external websites referred to in this publication. C. M. Manaia acknowledges National Funds from FCT—Fundação para a Ciência e a Tecnologia through projects PEst-OE/EQB/LA0016/2011 and PTDC/AAC-AMB/113840/2009. F. Walsh acknowledges funding from the Swiss Federal Office for Agriculture, the Swiss Federal Office for the Environment and the Swiss Expert Committee for Biosafety (SECB). Finally L. Cantas extend his thanks to MegaVet.no, for helpful support in reviewing the manuscript.

### Conflict of interest statement

The authors declare that the research was conducted in the absence of any commercial or financial relationships that could be construed as a potential conflict of interest.

## References

[B1] Aarestrup F. M. (2005). Veterinary drug usage and antimicrobial resistance in bacteria of animal origin. Basic Clin. Pharmacol. Toxicol. 96, 271–281 10.1111/j.1742-7843.2005.pto960401.x 15755309

[B2] Aarestrup F. M. Seyfarth A. M. Emborg H. D. Pedersen K. Hendriksen R. S. Bager F. (2001). Effect of abolishment of the use of antimicrobial agents for growth promotion on occurrence of antimicrobial resistance in fecal Enterococci from food animals in Denmark. Antimicrob. Agents Chemother. 45, 2054–2059 10.1128/AAC.45.7.2054-2059.2001 11408222 PMC90599

[B3] Abraham E. P. Chain E. (1940). An enzyme from bacteria able to destroy penicillin. Nature 146, 837 3055168

[B4] Agersø Y. Petersen A. (2007). The tetracycline resistance determinant Tet 39 and the sulphonamide resistance gene sulII are common among resistant Acinetobacter spp. isolated from integrated fish farms in Thailand. J. Antimicrob. Chemother. 59, 23–27 10.1093/jac/dkl419 17095527

[B5] Alanis A. J. (2005). Resistance to antibiotics: are we in the post-antibiotic era? Arch. Med. Res. 36, 697–705 10.1016/j.arcmed.2005.06.009 16216651

[B6] Alexander T. W. Yanke J. L. Reuter T. Topp E. Read R. R. Selinger B. L. (2011). Longitudinal characterization of antimicrobial resistance genes in feces shed from cattle fed different subtherapeutic antibiotics. BMC Microbiol. 11:19 10.1186/1471-2180-11-19 21261985 PMC3037836

[B7] Allen H. K. Donato J. Wang H. H. Cloud-Hansen K. A. Davies J. Handelsman J. (2010). Call of the wild: antibiotic resistance genes in natural environments. Nat. Rev. Microbiol. 8, 251–259 10.1038/nrmicro2312 20190823

[B8] American Academy of Microbiology. (2009). Antibiotic resistance: an ecological perspective on an old problem, in Report of a colloquium, 12 to 14 October 2008 (Annecy, France). 32644325

[B9] Aminov R. Mackie R. I. (2007). Evolution and ecology of antibiotic resistance genes. FEMS Microbiol. Lett. 271, 147–161 10.1111/j.1574-6968.2007.00757.x 17490428

[B10] Anderson A. D. Nelson J. M. Rossiter S. Angulo F. J. (2003). Public health consequences of use of antimicrobial agents in food animals in the United States. Microb. Drug Resist. 9, 373–379 10.1089/107662903322762815 15000744

[B11] Angulo F. J. Nargund V. N. Chiller T. C. (2004). Evidence of an association between use of anti-microbial agents in food animals and anti-microbial resistance among bacteria isolated from humans and the human health consequences of such resistance. J. Vet. Med. B 51, 374–379 10.1111/j.1439-0450.2004.00789.x 15525369

[B12] Anthony F. Acar J. Franklin A. Gupta R. Nicholls T. Tamura Y. (2001). Antimicrobial resistance: responsible and prudent use of antimicrobial agents in veterinary medicine. Rev. Sci. Tech. 20, 829–839 11732425 10.20506/rst.20.3.1318

[B13] Arya S. C. Agarwal N. (2011). International travel with acquisition of multi-drug resistant Gram negative bacteria containing the New Delhi metallo-beta-lactamase gene, bla(NDM-1). Travel Med. Infect. Dis. 9, 47–48 10.1016/j.tmaid.2010.12.002 21269886

[B14] Ash R. J. Mauck B. Morgan M. (2002). Antibiotic resistance of gram-negative bacteria in rivers, United States. Emerg. Infect. Dis. 8, 713–716 10.3201/eid0807.010264 12095440 PMC2730334

[B15] Baltz R. H. (2007). Antimicrobials from actinomycetes: back to the future. Microbe 2, 125–131

[B16] Bannoehr J. Ben Zakour N. L. Waller A. S. Guardabassi L. Thoday K. L. van den Broek A. H. (2007). Population genetic structure of the *Staphylococcus intermedius* group: insights into agr diversification and the emergence of methicillin-resistant strains. J. Bacteriol. 189, 8685–8692 10.1128/JB.01150-07 17905991 PMC2168937

[B17] Baquero F. Alvarez-Ortega C. Martinez J. L. (2009). Ecology and evolution of antibiotic resistance. Environ. Microbiol. Rep. 1, 469–476 10.1111/j.1758-2229.2009.00053.x 23765924

[B18] Baquero F. Martinez J.-L. Canton R. (2008). Antibiotics and antibiotic resistance in water environments. Curr. Opin. Biotechnol. 19, 260–265 10.1016/j.copbio.2008.05.006 18534838

[B19] Barber M. Rozwadowska-Dowzenko M. (1948). Infection by penecillin-resistant staphylococci. Lancet 2, 641–644 18890505 10.1016/s0140-6736(48)92166-7

[B20] Bastianello S. S. Fourie N. Prozesky L. Nel P. W. Kellermann T. S. (1995). Cardiomyopathy of ruminants induced by the litter of poultry fed on rations containing the ionophore antibiotic, maduramicin.2. macropathology and histopathology. Onderstepoort J. Vet. Res. 62, 5–18 8539035

[B21] Björklund H. V. Råbergh C. M. I. Bylund G. (1991). Residues of oxolinic acid and oxytetracycline in fish and sediments from fish farms. Aquaculture 97, 85–96

[B22] Blanch A. R. Caplin J. L. Iversen A. Kühn I. Manero A. Taylor H. D. (2003). Comparison of enterococcal populations related to urban and hospital wastewater in various climatic and geographic European regions. J. Appl. Microbiol. 94, 994–1002 10.1046/j.1365-2672.2003.01919.x 12752807

[B23] Böckelmann U. Dorries H. H. Ayuso-Gabella M. N. Marçay M. S. Tandoi V. Levantesi C. (2009). Quantitative PCR monitoring of antibiotic resistance genes and bacterial pathogens in three European artificial groundwater recharge systems. J. Appl. Environ. Microbiol. 75, 154–163 10.1128/AEM.01649-08 19011075 PMC2612202

[B24] Brown S. Bantar C. Young H. K. Amyes S. G. B. (1998). Limitation of *Acinetobacter baumannii* treatment by plasmid-mediated carbapenemase ARI-2. Lancet 351, 186–187 10.1016/S0140-6736(05)78210-6 9449879

[B25] Bush K. Courvalin P. Dantas G. Davies J. Eisenstein B. Huovinen P. (2011). Tackling antibiotic resistance. Nat. Rev. Microbiol. 9, 894–896 10.1038/nrmicro2693 22048738 PMC4206945

[B26] Cabello F. C. (2006). Heavy use of prophylactic antibiotics in aquaculture: a growing problem for human and animal health and for the environment. Environ. Microbiol. 8, 1137–1144 10.1111/j.1462-2920.2006.01054.x 16817922

[B27] Callaway T. R. Edrington T. S. Anderson R. C. Harvey R. B. Genovese K. J. Kennedy C. N. (2008). Probiotics, prebiotics and competitive exclusion for prophylaxis against bacterial disease. Anim. Health Res. Rev. 9, 217–225 10.1017/S1466252308001540 19102792

[B28] Cantas L. Fraser T. W. K. Fjelldal P. G. Mayer I. Sørum H. (2011). The culturable intestinal microbiota of triploid and diploid juvenile Atlantic salmon (*Salmo salar*)—a comparison of composition and drug resistance. BMC Vet. Res. 7:71 10.1186/1746-6148-7-71 22094054 PMC3239839

[B29] Cantas L. Le Roux F. Mazel D. Sørum H. (2012a). Impact of Antibiotics on the Expression of the tra Genes and on the Host Innate Immune Gene Activity during SXT Element Bearing Aeromonas salmonicida Infection in Atlantic Salmon (Salmo salar L.). PSN-ICAAC12PS1-B-1330. San Francisco, CA

[B30] Cantas L. Torp J. R. Alestrøm P. Sørum H. (2012b). Cultureable gut microbiota diversity in zebrafish. Zebrafish 9, 26–37 10.1089/zeb.2011.0712 22428747 PMC3308716

[B31] Cantas L. Midtlyng P. J. Sørum H. (2012c). Impact of antibiotic treatments on the expression of the R plasmid tra genes and on the host innate immune activity during pRAS1 bearing *Aeromonas hydrophila* infection in zebrafish (*Danio rerio*). BMC Microbiol. 12:37 10.1186/1471-2180-12-37 22429905 PMC3340321

[B32] Cantas L. Thoresen I. S. Gjermund G. Sivertsen T. Framstad T. Sørum H. (2013). Antibiotikabehandling av kolidiaré hos spedgris med minimal antibiotikaresistensutvikling—en modellstudie (in Norwegian), in Husdyrforsøksmøte 2013 - UMB (Lillestrøm).

[B33] Caprioli A. Busani L. Martel J. L. Helmuth R. (2000). Monitoring of antibiotic resistance in bacteria of animal origin: epidemiological and microbiological methodologies. Int. J. Antimicrob. Agents 14, 295–301 10794950 10.1016/s0924-8579(00)00140-0

[B34] Casewell M. Friis C. Marco E. McMullin P. Phillips I. (2003). The European ban on growth-promoting antibiotics and emerging consequences for human and animal health. J. Antimicrob. Chemother. 52, 159–161 10.1093/jac/dkg313 12837737

[B35] Cattoir V. Poirel L. Mazel D. Soussy C. J. Nordmann P. (2007). *Vibrio splendidus* as the source of plasmid-mediated qnrS-Like quinolone resistance determinants. Antimicrob. Agents Chemother. 51, 2650–2651 10.1128/AAC.00070-07 17452482 PMC1913262

[B37] Cheng A. C. Turnidge J. Collignon P. Looke D. Barton M. Gottlieb T. (2012). Control of fluoroquinolone resistance through successful regulation, Australia. Emerg. Infect. Dis. 18, 1453–1460 10.3201/eid1809.111515 22932272 PMC3437704

[B38] Colquhoun D. J. Aarflot L. Melvold C. F. (2007). gyrA and parC mutations and associated quinolone resistance in *Vibrio anguillarum* serotype O2b strains isolated from farmed Atlantic cod (*Gadus morhua*) in Norway. Antimicrob. Agents Chemother. 51, 2597–2599 10.1128/AAC.00315-07 17502408 PMC1913251

[B39] Corpet D. E. (1988). Antibiotic resistance from food. N. Engl. J. Med. 318, 1206–1207 3362171 10.1056/nejm198805053181818 PMC2814222

[B40] Crofton J. Mitchison D. A. (1948). Streptomycin resistance in pulmonary tuberculosis. Br. Med. J. 2, 1009–1015 18100441 10.1136/bmj.2.4588.1009 PMC2092236

[B41] Cunha B. A. (2002). Strategies to control antibiotic resistance. Semin. Respir. Infect. 17, 250–258 12226805 10.1053/srin.2002.34692

[B42] Cuny C. Friedrich A. Kozytska S. Layer F. Nubel U. Ohlsen K. (2010). Emergence of methicillin-resistant *Staphylococcus aureus* (MRSA) in different animal species. Int. J. Med. Microbiol. 300, 109–117 10.1016/j.ijmm.2009.11.002 20005777

[B43] Czekalski N. Berthold T. Caucci S. Egli A. Burgmann H. (2012). Increased levels of multiresistant bacteria and resistance genes after wastewater treatment and their dissemination into Lake Geneva, Switzerland. Front. Microbiol. 3:106 10.3389/fmicb.2012.00106 22461783 PMC3310248

[B44] D'Costa V. M. King C. E. Kalan L. Morar M. Sung W. W. L. Schwarz C. (2011). Antibiotic resistance is ancient. Nature 477, 457–461 10.1038/nature10388 21881561

[B45] D'Costa V. M. McGrann K. M. Hughes D. W. Wright G. D. (2006). Sampling the antibiotic resistome. Science 311, 374–377 10.1126/science.1120800 16424339

[B46] Decousser J. W. Poirel L. Nordmann P. (2001). Characterization of a chromosomally encoded extended-spectrum class A β-lactamase from *Kluyvera cryocrescens*. Antimicrob. Agents Chemother. 45, 3595–3598 10.1128/AAC.45.12.3595-3598.2001 11709346 PMC90875

[B47] Defoirdt T. Sorgeloos P. Bossier P. (2011). Alternatives to antibiotics for the control of bacterial disease in aquaculture. Curr. Opin. Microbiol. 14, 251–258 10.1016/j.mib.2011.03.004 21489864

[B48] Dodd M. C. (2012). Potential impacts of disinfection processes on elimination and deactivation of antibiotic resistance genes during water and wastewater treatment. J. Environ. Monit. 14, 1754–1771 10.1039/c2em00006g 22572858

[B49] Derewacz D. K. Goodwin C. R. McNees C. R. McLean J. A. Bachmann B. O. (2013). Antimicrobial drug resistance affects broad changes in metabolomic phenotype in addition to secondary metabolism. Proc. Natl. Acad. Sci. U.S.A. 110, 2336–2341 10.1073/pnas.1218524110 23341601 PMC3568320

[B50] FAAIR Scientific. (2002). Select findings and conclusions. Clin. Infect. Dis. 34, S73–S75 10.1086/340242 11988875

[B51] Falagas M. E. Bliziotis I. A. (2007). Pandrug-resistant Gram-negative bacteria: the dawn of the post-antibiotic era? Int. J. Antimicrob. Agents 29, 630–636 10.1016/j.ijantimicag.2006.12.012 17306965

[B52] Falcone-Dias M. F. Vaz-Moreira I. Manaia C. M. (2012). Bottled mineral water as a potential source of antibiotic resistant bacteria. Water Res. 46, 3612–3622 10.1016/j.watres.2012.04.007 22534119

[B53] FAO. (2010). The State of the World Fisheries and Aquaculture. Rome: United Nations Food and Agriculture Organization

[B54] Farmed Fish Health Report. (2010). Norwegian Veterinary Institute, in The Health Situation in Norwegian Aquaculture 2010, eds Bornø G. Sviland C. (Oslo: Norwegian Veterinary Institute).

[B55] Ferreira da Silva M. Tiago I. Verissimo A. Boaventura R. A. R. Nunes O. C. Manaia C. M. (2006). Antibiotic resistance of enterococci and related bacteria in an urban wastewater treatment plant. FEMS Microbiol. Ecol. 55, 322–329 10.1111/j.1574-6941.2005.00032.x 16420639

[B56] Ferreira da Silva M. Vaz-Moreira I. Gonzalez-Pajuelo M. Nunes O. C. Manaia C. M. (2007). Antimicrobial resistance patterns in Enterobacteriaceae isolated from an urban wastewater treatment plant. FEMS Microbiol. Ecol. 60, 166–176 10.1111/j.1574-6941.2006.00268.x 17250754

[B57] Filice G. A. Nyman J. A. Lexau C. Lees C. H. Bockstedt L. A. Como-Sabetti K. (2010). Excess costs and utilization associated with methicillin resistance for patients with *Staphylococcus aureus* infection. Infect. Control Hosp. Epidemiol. 31, 365–373 10.1086/651094 20184420

[B58] Fischer J. Rodriguez I. Schmoger S. Friese A. Roesler U. Helmuth R. (2012). *Escherichia coli* producing VIM-1 carbapenemase isolated on a pig farm. J. Antimicrob. Chemother. 67, 1793–1795 10.1093/jac/dks108 22454489

[B59] Forsberg K. J. Reyes A. Wang B. Selleck E. M. Sommer M. O. A. Dantas G. (2012). The shared antibiotic resistome of soil bacteria and human pathogens. Science 337, 1107–1111 10.1126/science.1220761 22936781 PMC4070369

[B60] Giguêre S. Prescott J. F. Baggot J. D. Walker R. D. Dowling P. M. (2007). Antimicrobial Therapy in Veterinary Medicine. Ames; Oxford: Iowa State University Press

[B61] Glad T. Bernhardsen P. Nielsen K. M. Brusetti L. Andersen M. Aars J. (2010). Bacterial diversity in faeces from polar bear (*Ursus maritimus*) in Arctic Svalbard. BMC Microbiol. 10:10 10.1186/1471-2180-10-10 20074323 PMC2822771

[B62] Goñi-Urriza M. Capdepuy M. Arpin C. Raymond N. Caumette P. Quentin C. (2000). Impact of an urban effluent on antibiotic resistance of riverine *Enterobacteriaceae* and *Aeromonas* spp. J. Appl. Environ. Microb. 66, 125–132 10.1128/AEM.66.1.125-132.2000 10618213 PMC91795

[B62a] Götz A. Pukall R. Smit E. Tietze E. Prager R. Tschäpe H. (1996). Detection and characterization of broad-host range plasmids in environmental bacteria by PCR. Appl. Environ. Microbiol. 62, 2621–2628 8779598 10.1128/aem.62.7.2621-2628.1996 PMC168041

[B63] Graham D. W. Olivares-Rieumont S. Knapp C. W. Lima L. Werner D. Bowen E. (2011). Antibiotic resistance gene abundances associated with waste discharges to the Almendares River near Havana, Cuba. Environ. Sci. Technol. 45, 418–424 10.1021/es102473z 21133405 PMC3024002

[B64] Gräslund S. Holmström K. Wahlström A. (2003). A field survey of chemicals and biological products used in shrimp farming. Mar. Pollut. Bull. 46, 81–90 10.1016/S0025-326X(02)00320-X 12535973

[B65] Grave K. Torren-Edo J. Mackay D. (2010). Comparison of the sales of veterinary antibacterial agents between 10 European countries. J. Antimicrob. Chemother. 65, 2037–2040 10.1093/jac/dkq247 20587611

[B66] Gullberg E. Cao S. Berg O. G. Ilback C. Sandegren L. Hughes D. (2011). Selection of resistant bacteria at very low antibiotic concentrations. PLoS Pathog. 7:e1002158 10.1371/journal.ppat.1002158 21811410 PMC3141051

[B67] Gustafson R. H. Bowen R. E. (1997). Antibiotic use in animal agriculture. J. Appl. Microbiol. 83, 531–541 9418018 10.1046/j.1365-2672.1997.00280.x

[B68] Halverson M. (2000). The Price We Pay for Corporate Hogs. Minneapolis, MN: Institute for Agriculture and Trade policy, 32

[B69] Hammerum A. M. Heuer O. E. Emborg H. D. Bagger-Skjot L. Jensen V. F. Rogues A. M. (2007). Danish integrated antimicrobial in resistance monitoring and research program. Emerg. Infect. Dis. 13, 1632–1639 10.3201/eid1311.070421 18217544 PMC3375779

[B70] Hektoen H. Berge J. A. Hormazabal V. Yndestad M. (1995). Persistence of antibacterial agents in marine sediments. Aquaculture 133, 175–184

[B71] Heuer O. E. Kruse H. Grave K. Collignon P. Karunasagar I. Angulo F. J. (2009). Human health consequences of use of antimicrobial agents in aquaculture. Clin. Infect. Dis. 49, 1248–1253 10.1086/605667 19772389

[B72] Hoa P. T. P. Managaki S. Nakada N. Takada H. Shimizu A. Anh D. H. (2011). Antibiotic contamination and occurrence of antibiotic-resistant bacteria in aquatic environments of northern Vietnam. Sci. Total Environ. 409, 2894–2901 10.1016/j.scitotenv.2011.04.030 21669325

[B73] Holmberg S. D. Solomon S. L. Blake P. A. (1987). Health and economic impacts of antimicrobial resistance. Rev. Infect. Dis. 9, 1065–1078 3321356 10.1093/clinids/9.6.1065

[B74] Holmström K. Gräslund S. Wahlström A. Poungshompoo S. Bengtsson B. E. Kautsky N. (2003). Antibiotic use in shrimp farming and implications for environmental impacts and human health. Int. J. Food Sci. Technol. 38, 255–266

[B75] Horton R. A. Randall L. P. Snary E. L. Cockrem H. Lotz S. Wearing H. (2011). Fecal carriage and shedding density of CTX-M extended-spectrum β-lactamase-producing *Escherichia coli* in cattle, chickens, and pigs: implications for environmental contamination and food production. Appl. Environ. Microbiol. 77, 3715–3719 10.1128/AEM.02831-10 21478314 PMC3127594

[B76] Jean-Louis M. Tardy F. Brisabois A. Lailler R. Coudert M. Chaslus-Dancla E. (2000). The French antibiotic resistance monitoring programs. Int. J. Antimicrob. Agents 14, 275–283 10794947 10.1016/s0924-8579(00)00137-0

[B78] Klugman K. P. (2002). Bacteriological evidence of antibiotic failure in pneumococcal lower respiratory tract infections. Eur. Respir. J. 20, 3S–8S 10.1183/09031936.02.00400402 12168746

[B79] Kluytmans J. A. J. W. (2010). Methicillin-resistant *Staphylococcus aureus* in food products: cause for concern or case for complacency? Clin. Microbiol. Infect. 16, 11–15 10.1111/j.1469-0691.2009.03110.x 20002686

[B80] Knapp C. W. Dolfing J. Ehlert P. A. Graham D. W. (2010). Evidence of increasing antibiotic resistance gene abundances in archived soils since 1940. Environ. Sci. Technol. 44, 580–587 10.1021/es901221x 20025282

[B81] Knapp C. W. Engemann C. A. Hanson M. L. Keen P. L. Hall K. J. Graham D. W. (2008). Indirect evidence of transposon-mediated selection of antibiotic resistance genes in aquatic systems at low-level oxytetracycline exposures. Environ. Sci. Technol. 42, 5348–5353 18754392 10.1021/es703199g

[B82] Kohanski M. A. DePristo M. A. Collins J. J. (2010). Sublethal antibiotic treatment leads to multidrug resistance via radical-induced mutagenesis. Mol. Cell 37, 311–320 10.1016/j.molcel.2010.01.003 20159551 PMC2840266

[B83] Korczak D. Schöffmann C. (2010). Medical and health economic evaluation of prevention- and control measures related to MRSA infections or -colonisations at hospitals. GMS Health Technol. Assess. 6, 1–9 10.3205/hta000082 21289877 PMC3010887

[B84] Kumarasamy K. K. Toleman M. A. Walsh T. R. Bagaria J. Butt F. Balakrishnan R. (2010). Emergence of a new antibiotic resistance mechanism in India, Pakistan, and UK: a molecular, biological, and epidemiological study. Lancet Infect. Dis. 10, 597–602 10.1016/S1473-3099(10)70143-2 20705517 PMC2933358

[B87] Lang K. S. Anderson J. M. Schwarz S. Williamson L. Handelsman J. Singer R. S. (2010). Novel florfenicol and chloramphenicol resistance gene discovered in Alaskan soil by using functional metagenomics. Appl. Environ. Microbiol. 76, 5321–5326 10.1128/AEM.00323-10 20543056 PMC2916469

[B88] Levy S. B. (1984). Antibiotic resistant bacteria in food of man and animals, in Antimicrobials Ans Agriculture, ed Woodbine M. (London: Butterworths), 525–531

[B89] Levy S. B. (1998). The challange of antibiotic resistance. Sci. Am. 278, 46–53 9487702 10.1038/scientificamerican0398-46

[B90] Levy S. B. (2001). Antibiotic resistance: consequences of inaction. Clin. Infect. Dis. 33, 124–129 10.1086/321837 11524708

[B91] Levy S. B. FitzGerald G. B. Macone A. B. (1976). Changes in intestinal flora of farm personnel after introduction of a tetracycline-supplemented feed on a farm. N. Engl. J. Med. 295, 583–588 10.1056/NEJM197609092951103 950974

[B92] Levy S. B. Marshall B. (2004). Antibacterial resistance worldwide: causes, challenges and responses. Nat. Med. 10, 122–129 10.1038/nm1145 15577930

[B93] Lie Ø. (2008). Improving Farmed Fish Quality and Safety. Woodhead, Publishing Series in Food Science, Technology and Nutrition No. 162. E-ISBN: 978-1-84569-492-0.

[B94] Linton K. B. Lee P. A. Rowland A. J. Baker V. N. Gillespi W. A. Richmond M. H. (1972). Antibiotic resistance and transmissible R-factors in intestinal Coliform flora of healthy adults and children in an urban and a rural community. J. Hyg. (Lond.) 70, 99–104 4552258 10.1017/s0022172400022130 PMC2130013

[B95] Literak I. Petro R. Dolejska M. Gruberova E. Dobiasova H. Petr J. (2011). Antimicrobial resistance in fecal *Escherichia coli* isolates from healthy urban children of two age groups in relation to their antibiotic therapy. Antimicrob. Agents Chemother. 55, 3005–3007 10.1128/AAC.01724-10 21464246 PMC3101410

[B96] Ma J. Liu J. H. Lv L. Zong Z. Sun Y. Zheng H. (2012). Characterization of extended-spectrum β-lactamase genes among *Escherichia coli* isolates from duck and environment samples in a duck farm. Appl. Environ. Microbiol. 78, 3668–3673 10.1128/AEM.07507-11 22407683 PMC3346353

[B97] Markestad A. Grave K. (1997). Reduction of antibacterial drug use in Norwegian fish farming due to vaccination. Dev. Biol. Stand. 90, 365–369 9270865

[B98] Marshall B. Petrowski D. Levy S. B. (1990). Inter- and intraspecies spread of *Escherichia coli* in a farm environment in the absence of antibiotic usage. Proc. Natl. Acad. Sci. U.S.A. 87, 6609–6613 2204058 10.1073/pnas.87.17.6609 PMC54586

[B99] Marshall B. M. Levy S. B. (2011). Food animals and antimicrobials: impacts on human health. Clin. Microbiol. Rev. 24, 718–733 10.1128/CMR.00002-11 21976606 PMC3194830

[B100] Martinez J. L. (2009). Environmental pollution by antibiotics and by antibiotic resistance determinants. Environ. Pollut. 157, 2893–2902 10.1016/j.envpol.2009.05.051 19560847

[B101] McEwen S. A. (2006). Antibiotic use in animal agriculture: what have we learned and where are we going? Anim. Biotechnol. 17, 239–250 10.1080/10495390600957233 17127534

[B102] McManus P. S. Stockwell V. O. Sundin G. W. Jones A. L. (2002). Antibiotic use in plant agriculture. Annu. Rev. Phytopathol. 40, 443–465 10.1146/annurev.phyto.40.120301.093927 12147767

[B103] Michael I. Rizzo L. McArdell C. S. Manaia C. M. Merlin C. Schwartz T. (2013). Urban wastewater treatment plants as hotspots for the release of antibiotics in the environment: a review. Water Res. 47, 957–995 10.1016/j.watres.2012.11.027 23266388

[B104] Midtlyng P. J. Grave K. Horsberg T. E. (2011). What has been done to minimize the use of antibacterial and antiparasitic drugs in Norwegian aquaculture. Aquac. Res. 42, 28–34

[B105] Moura A. Henriques I. Smalla K. Correia A. (2010). Wastewater bacterial communities bring together broad-host range plasmids, integrons and a wide diversity of uncharacterized gene cassettes. Res. Microbiol. 161, 58–66 10.1016/j.resmic.2009.11.004 20004718

[B106] Muto C. A. Jernigan J. A. Ostrowsky B. E. Richet H. M. Jarvis W. R. Boyce J. M. (2003). SHEA guideline for preventing nosocomial transmission of multidrug-resistant strains of *Staphylococcus aureus* and Enterococcus. Infect. Control Hosp. Epidemiol. 24, 362–386 10.1086/502213 12785411

[B107] Ndi O. Barton M. (2012). Antibiotic resistance in animals—The Australian perspective, in Antimicrobial Resistance in the Environment, eds Keen P. L. Montforts M. H. H. M. (New Jersey, NJ: Wiley-Blackwell), 265–290

[B108] Nkogwe C. Raletobana J. Stewart-Johnson A. Suepaul S. Adesiyun A. (2011). Frequency of detection of *Escherichia coli*, Salmonella spp., and Campylobacter spp. in the faeces of wild rats (Rattus spp.) in Trinidad and Tobago. Vet. Med. Int. 2011:686923 10.4061/2011/686923 21547220 PMC3087471

[B109] Nordmann P. Dortet L. Poirel L. (2012). Carbapenem resistance in Enterobacteriaceae: here is the storm! Trends Mol. Med. 18, 263–272 10.1016/j.molmed.2012.03.003 22480775

[B110] Nordmann P. Poirel L. Toleman M. A. Walsh T. R. (2011). Does broad-spectrum β-lactam resistance due to NDM-1 herald the end of the antibiotic era for treatment of infections caused by Gram-negative bacteria? J. Antimicrob. Chemother. 66, 689–692 10.1093/jac/dkq520 21393184

[B111] NORM/NORM-VET. (2011). Usage of Antimicrobial Agents and Occurrence of Antimicrobial Resistance in Norway. Tromsø/Oslo 2012. ISSN:1502-2307 (print)/1890-9965 (electronic). (Tromsø/Oslo: Norwegian Veterinary Institute). Available online at: http://www.vetinst.no/eng/Publications/Norm-Norm-Vet-Report/Norm-Norm-Vet-rapporten-2010

[B112] Novo A. André S. Viana P. Nunes O. C. Manaia C. M. (2013). Antibiotic resistance, antimicrobial residues and bacterial community composition in urban wastewater. Water Res. 47, 1875–1887 10.1016/j.watres.2013.01.010 23375783

[B113] Novo A. Manaia C. M. (2010). Factors influencing antibiotic resistance burden in municipal wastewater treatment plants. Appl. Microbiol. Biotechnol. 87, 1157–1166 10.1007/s00253-010-2583-6 20396880

[B114] Oberlé K. Capdeville M. Berthe T. Petit F. (2012). Evidence for a complex relationship between antibiotics and antibiotic-resistant *Escherichia coli*: from medical center patients to a receiving environment. Environ. Sci. Technol. 46, 1859–1868 10.1021/es203399h 22216912

[B115] Olarte J. (1983). Antibiotic resistance in Mexico. APUA Newslett. 1, 3

[B116] Olliver A. Vallê M. Chaslus-Dancla E. Cloeckaert A. (2005). Overexpression of the multidrug efflux operon acrEF by insertional activation with IS1 or IS10 elements in *Salmonella enterica* Serovar *Typhimurium* DT204 acrB mutants selected with fluoroquinolones. Antimicrob. Agents Chemother. 49, 289–301 10.1128/AAC.49.1.289-301.2005 15616308 PMC538886

[B117] Otter J. A. French G. L. (2010). Molecular epidemiology of community-associated meticillin-resistant *Staphylococcus aureus* in Europe. Lancet Infect. Dis. 10, 227–239 10.1016/S1473-3099(10)70053-0 20334846

[B118] Pallett A. Hand K. (2010). Complicated urinary tract infections: practical solutions for the treatment of multiresistant Gram-negative bacteria. J. Antimicrob. Chemother. 65, 25–33 10.1093/jac/dkq298 20876625

[B119] Patra S. Das T. K. Avila C. Cabello V. Castillio F. Sarkar D. (2012). Cadmium tolerance and antibiotic resistance in *Escherichia coli* isolated from waste stabilization ponds. Indian J. Exp. Biol. 50, 300–307 22611919

[B120] Petersen A. Andersen J. S. Kaewmak T. Somsiri T. Dalsgaard A. (2002). Impact of integrated fish farming on antimicrobial resistance in a pond environment. Appl. Environ. Microbiol. 68, 6036–6042 10.1128/AEM.68.12.6036-6042.2002 12450826 PMC134385

[B121] Picao R. C. Poirel L. Demarta A. Silva C. S. F. Corvaglia A. R. Petrini O. (2008). Plasmid-mediated quinolone resistance in *Aeromonas allosaccharophila* recovered from a Swiss lake. J. Antimicrob. Chemother. 62, 948–950 10.1093/jac/dkn341 18772162

[B122] Poirel L. Liard A. Rodriguez-Martinez J. M. Nordmann P. (2005). Vibrionaceae as a possible source of Qnr-like quinolone resistance determinants. J. Antimicrob. Chemother. 56, 1118–1121 10.1093/jac/dki371 16227349

[B123] Popowska M. Krawczyk-Balska A. (2013). Broad-Host-Range IncP-1 plasmids and their resistance potential. Front. Microbiol. 4:44 10.3389/fmicb.2013.00044 23471189 PMC3590792

[B124] Popowska M. Miernik A. Rzeczycka M. Lopaciuk A. (2010). The impact of environmental contamination with antibiotics on levels of resistance in soil bacteria. J. Environ. Qual. 39, 1679–1687 21043273 10.2134/jeq2009.0499

[B125] Popowska M. Rzeczycka M. Miernik A. Krawczyk-balska A. Walsh F. Duffy B. (2012). Influence of soil use on prevalence of tetracycline, streptomycin, and erythromycin resistance and associated resistance genes. Antimicrob. Agents Chemother. 56, 1434–1443 10.1128/AAC.05766-11 22203596 PMC3294877

[B126] Potter A. Gerdts V. Littel-Van den Hurk S. V. (2008). Veterinary vaccines: alternatives to antibiotics? Anim. Health Res. Rev. 9, 187–199 10.1017/S1466252308001606 19102790

[B127] Pouillard J. (2002). A forgotten discovery: doctor of medicine Ernest Duchesne, s thesis (1874-1912) [Article in French]. Hist. Sci. Med. 36, 11–20 12094813

[B128] Pratt R. (2010). Preparation for a post antibiotic era must start now. Nurs. Times 106, 26 21086836

[B129] Primavera J. H. (2006). Overcoming the impacts of aquaculture on the coastal zone. Ocean Coast. Manage. 49, 531–545

[B130] Pruden A. Arabi M. Storteboom H. N. (2012). Correlation between upstream human activities and riverine antibiotic resistance genes. Environ. Sci. Technol. 46, 11541-11549 10.1021/es302657r 23035771

[B131] Rassow D. Schaper H. (1996). The use of feed medications in swine and poultry facilities in the Weser-Ems region [Article in German]. Dtsch. Tierarztl. Wochenschr. 103, 244–249 8998936

[B132] Reinthaler F. F. Posch J. Feierl G. Wüst G. Haas D. Ruckenbauer G. (2003). Antibiotic resistance of *E. coli* in sewage and sludge. Water Res. 37, 1685–1690 10.1016/S0043-1354(02)00569-9 12697213

[B133] Rhodes G. Huys G. Swings J. McGann P. Hiney M. Smith P. (2000). Distribution of oxytetracycline resistance plasmids between Aeromonads in hospital and aquaculture environments: implication of Tn1721 in dissemination of the tetracycline resistance determinant Tet, A. Appl. Environ. Microbiol. 66, 3883–3890 10.1128/AEM.66.9.3883-3890.2000 10966404 PMC92234

[B134] Riesenfeld C. S. Goodman R. M. Handelsman J. (2004a). Uncultured soil bacteria are a reservoir of new antibiotic resistance genes. Environ. Microbiol. 6, 981–989 10.1111/j.1462-2920.2004.00664.x 15305923

[B134b] Riesenfeld C. S. Schloss P. D. Handelsman J. (2004b). Metagenomics: genomic analysis of microbial communities. Annu. Rev. Genet. 38, 525–552 10.1146/annurev.genet.38.072902.091216 15568985

[B135] Rizzo L. Manaia C. M. Merlin C. Schwartz T. Dagot D. Ploy M. C. (2013). Urban wastewater treatment plants as hotspots for antibiotic resistant bacteria and genes spread into the environment: a review. Sci. Total Environ. 447, 345–360 10.1016/j.scitotenv.2013.01.032 23396083

[B136] Roberts R. R. Hota B. Ahmad I. Scott R. D. Foster S. D. Abbasi F. (2009). Hospital and societal costs of antimicrobial-resistant infections in a Chicago teaching hospital: implications for antibiotic stewardship. Clin. Infect. Dis. 49, 1175–1184 10.1086/605630 19739972

[B137] Rodriguez M. M. Power P. Radice M. Vay C. Famiglietti A. Galleni M. (2004). Chromosome-encoded CTX-M-3 from *Kluyvera ascorbata*: a possible origin of plasmid-borne CTX-M-1-derived cefotaximases. Antimicrob. Agents Chemother. 48, 4895–4897 10.1128/AAC.48.12.4895-4897.2004 15561876 PMC529199

[B138] Roe M. T. Pillai S. D. (2003). Monitoring and identifying antibiotic resistance mechanisms in bacteria. Poult. Sci. 82, 622–626 12710483 10.1093/ps/82.4.622

[B139] Roura E. Homedes J. Klasing K. C. (1992). Prevention of immunologic stress contributes to the growth-permitting ability of dietary antibiotics in chicks. J. Nutr. 122, 2383–2390 1453223 10.1093/jn/122.12.2383

[B140] Sato K. Bartlett P. C. Saeed M. A. (2005). Antimicrobial susceptibility of *Escherichia coli* isolates from dairy farms using organic versus conventional production methods. J. Am. Vet. Med. Assoc. 226, 589–594 15742702 10.2460/javma.2005.226.589

[B141] Schwartz T. Volkmann H. Kirchen S. Kohnen W. Schön-Hölz K. Jansen B. (2006). Real-time PCR detection of *Pseudomonas aeruginosa* in clinical and municipal wastewater and genotyping of the ciprofloxacin-resistant isolates. FEMS Microbiol. Ecol. 57, 158–167 10.1111/j.1574-6941.2006.00100.x 16819959

[B142] Segura P. A. Francois M. Gagnon C. Sauve S. (2009). Review of the occurrence of anti-infectives in contaminated wastewaters and natural and drinking waters. Environ. Health Perspect. 117, 675–684 10.1289/ehp.11776 19479007 PMC2685827

[B143] Seiler C. Berendonk T. U. (2012). Heavy metal driven co-selection of antibiotic resistance in soil and water bodies impacted by agriculture and aquaculture. Front. Microbiol. 10.3389/fmicb.2012.00399 23248620 PMC3522115

[B144] Sen D. Van Der Auwera G. Rogers L. Thomas C. M. Brown C. J. Top E. M. (2011). Broad-host-range plasmids from agricultural soils have IncP-1 backbones with diverse accessory genes. Appl. Environ. Microbiol. 77, 7975–7983 10.1128/AEM.05439-11 21948829 PMC3209000

[B145] Serrano P. H. (2005). Responsible use of antibiotics in aquaculture, in FAO Fisheries technical paper 469 (Rome; United Nations), 89 Available online at: ftp://ftp.fao.org/docrep/fao/009/a0282e/a0282e00.pdf

[B146] Shah S. Q. A. Karatas S. Nilsen H. Steinum T. M. Colquhoun D. J. Sørum H. (2012a). Characterization and expression of the gyrA gene from quinolone resistant *Yersinia ruckeri* strains isolated from Atlantic salmon (*Salmo salar* L.) in Norway. Aquaculture 350–353, 37–41

[B147] Shah S. Q. A. Colquhoun D. J. Nikuli H. L. Sørum H. (2012b). Prevalence of antibiotic resistance genes in the bacterial flora of integrated fish farming environments of Pakistan and Tanzania. Environ. Sci. Technol. 46, 8672–8679 10.1021/es3018607 22823142

[B148] Sharma R. Munns K. Alexander T. Entz T. Mirzaagha P. Yanke L. J. (2008). Diversity and distribution of commensal fecal *Escherichia coli* bacteria in beef cattle administered selected subtherapeutic antimicrobials in a feedlot setting. Appl. Environ. Microbiol. 74, 6178–6186 10.1128/AEM.00704-08 18723654 PMC2570304

[B149] Shtienberg D. Zilberstaine M. Oppenheim D. Herzog Z. Manulis S. Shwartz H. (2001). Efficacy of oxolinic acid and other bactericides in suppression of *Erwinia amylovora* in pear orchards in Israel. Phytoparasitica 29, 143–154

[B150] Smith P. R. Le Breton A. Horsberg T. E. Corsin F. (2009). Guidelines for antimicrobial use in aquaculture, in Guide to Antimicrobial Use in Animals, eds Guardabassi L. Jensen L. B. Kruse H. (Oxford: Blackwell Publishing Ltd.), 207–218

[B151a] Soonthornchaikul N. Garelick H. Jones H. Jacobs J. Ball D. Choudhury M. (2006). Resistance to three antimicrobial agents of *Campylobacter* isolated from intensively and organically reared chickens purchased from retail outlets. Int. J. Antimicrob. Agents 27, 125–130 10.1016/j.ijantimicag.2005.09.020 16417991

[B151] Soonthornchaikul N. Garelick H. (2009). Antimicrobial resistance of Campylobacter species isolated from edible bivalve molluscs purchased from Bangkok markets, Thailand. Foodborne Pathog. Dis. 6, 947–951 10.1089/fpd.2008.0236 19622033

[B152] Sørum H. (2006). Antimicrobial drug resistance in fish pathogens, in Antimicrobial Resistance in Bacteria of Animal Origin, ed Aarestrup F. M. (Washington, DC: ASM Press), 213–238

[B153] Sørum H. (2008). Antibiotic resistance associated with veterinary drug use in fish farms, in Improving Farmed Fish Quality and Safety, ed Lie ø. (Cambridge: WoodHead Publishing Limit), 157–182

[B154] Stockwell V. O. Duffy B. (2012). Use of antibiotics in plant agriculture. Rev. Sci. Tech. 31, 199–210 22849276 10.20506/rst.31.1.2104

[B155] Stokes H. W. Gillings M. R. (2011). Gene flow, mobile genetic elements and the recruitment of antibiotic resistance genes into Gram negative pathogens. FEMS Microbiol. Rev. 35, 790–819 10.1111/j.1574-6976.2011.00273.x 21517914

[B156] Strahilevitz J. Jacoby G. Hooper D. C. Robicsek A. (2009). Plasmid-mediated quinolone resistance: a multifaceted threat. Clin. Microbiol. Rev. 22, 664–689 10.1128/CMR.00016-09 19822894 PMC2772364

[B157] Sundin G. W. Monks D. E. Bender C. L. (1995). Distribution of the streptomycin-resistance transposon Tn5393 among phylloplane and soil bacteria from managed agricultural habitats. Can. J. Microbiol. 41, 792–799 7585356 10.1139/m95-109

[B158] Swann M. M. (1969). Use of Antibiotics in Animal Husbandry and Veterinary Medicine (Swann Report). London: HMSO

[B159] Swartz M. N. (2002). Human diseases caused by foodborne pathogens of animal origin. Clin. Infect. Dis. 34, S111–S122 10.1086/340248 11988881

[B160] Szczepanowski R. H. Carpenter M. A. Czapinska H. Zaremba M. Tamulaitis G. Siksnys V. (2008). Central base pair flipping and discrimination by PspGI. Nucleic Acids Res. 36, 6109–6117 10.1093/nar/gkn622 18829716 PMC2577326

[B161] Szczepanowski R. Linke B. Krahn I. Gartemann K.-H. Gutzkow T. Eichler W. (2009). Detection of 140 clinically relevant antibiotic- resistance genes in the plasmid metagenome of wastewater treatment plant bacteria showing reduced susceptibility to selected antibiotics. Microbiology 155, 2306–2319 10.1099/mic.0.028233-0 19389756

[B36] Tablan O. C. Anderson L. J. Besser R. Bridges C. Hajjeh R. (2004). Guidelines for preventing health-care-associated pneumonia, 2003: recommendations of CDC and the Healthcare Infection Control Practices Advisory Committee. MMWR Recomm. Rep. 53, 1–36 15048056

[B162] Tennstedt T. Szczepanowski R. Braun S. Puhler A. Schluter A. (2003). Occurrence of integron-associated resistance gene cassettes located on antibiotic resistance plasmids isolated from a wastewater treatment plant. FEMS Microbiol. Ecol. 45, 239–252 10.1016/S0168-6496(03)00164-8 19719593

[B163] Thaker M. Spanogiannopoulos P. Wright G. D. (2010). The tetracycline resistome. Cell. Mol. Life Sci. 67, 419–431 10.1007/s00018-009-0172-6 19862477 PMC11115633

[B164] Thiele-Bruhn S. (2003). Pharmaceutical antibiotic compounds in soils—a review. J. Plant Nutr. Soil Sci. 166, 145–167

[B165] Torres-Cortés G. Millan V. Ramirez-Saad H. C. Nisa-Martinez R. Toro N. Martinez-Abarca F. (2011). Characterization of novel antibiotic resistance genes identified by functional metagenomics on soil samples. Environ. Microbiol. 13, 1101–1114 10.1111/j.1462-2920.2010.02422.x 21281423

[B166] Vaz-Moreira I. Nunes O. C. Manaia C. M. (2011). Diversity and antibiotic resistance patterns of Sphingomonadaceae isolated from drinking water. Appl. Environ. Microbiol. 77, 5697–5706 10.1128/AEM.00579-11 21705522 PMC3165245

[B167] Vazquez-Moreno L. Bermudez A. Langure A. Higuera-Ciapara I. Diaz De Aguayo M. lores E. (1990). Antibiotic residues and drug resistant bacteria in beef, and chicken tissues. J. Food Sci. 55, 632–634

[B168] Verschuere L. Rombaut G. Sorgeloos P. Verstraete W. (2000). Probiotic bacteria as biological control agents in aquaculture. Microbiol. Mol. Biol. Rev. 64, 655–671 10.1128/MMBR.64.4.655-671.2000 11104813 PMC99008

[B169] Volkmann H. Schwartz T. Bischoff P. Kirchen S. Obst U. (2004). Detection of clinically relevant antibiotic-resistance genes in municipal wastewater using real-time PCR (TaqMan). J. Microbiol. Methods 56, 277–286 10.1016/j.mimet.2003.10.014 14744456

[B170] Walsh T. R. Weeks J. Livermore D. M. Toleman M. A. (2011). Dissemination of NDM-1 positive bacteria in the New Delhi environment and its implications for human health: an environmental point prevalence study. Lancet Infect. Dis. 11, 355–362 10.1016/S1473-3099(11)70059-7 21478057

[B171] Watanabe T. (1963). Infective heredity of multiple drug resistance in bacteria. Bacteriol. Rev. 27, 87–115 13999115 10.1128/br.27.1.87-115.1963 PMC441171

[B172] Watkinson A. J. Micalizzi G. R. Bates J. R. Costanzo S. D. (2007). Novel method for rapid assessment of antibiotic resistance in *Escherichia coli* isolates from environmental waters by use of a modified chromogenic agar. Appl. Environ. Microbiol. 73, 2224–2229 10.1128/AEM.02099-06 17277213 PMC1855640

[B173] Weese J. S. (2010). Methicillin-resistant Staphylococcus aureus in animals. ILAR J. 51, 233–244 10.1093/ilar.51.3.233 21131724

[B174] Welch T. J. Fricke W. F. McDermott P. F. White D. G. Rosso M. L. Rasko D. A. (2007). Multiple antimicrobial resistance in plague: an emerging public health risk. PLoS ONE 2:e309 10.1371/journal.pone.0000309 17375195 PMC1819562

[B174a] Wieler L. H. Ewers C. Guenther S. Walther B. Lübke-Becker A. (2011). Methicillin-resistant staphylococci (MRS) and extended-spectrum beta-lactamases (ESBL)-producing *Enterobacteriaceae* in companion animals: nosocomial infections as one reason for the rising prevalence of these potential zoonotic pathogens in clinical samples. Int. J. Med. Microbiol. 301, 635–641 10.1016/j.ijmm.2011.09.009 22000738

[B175] Wilke M. H. (2010). Multiresistant bacteria and current therapy—the economical side of the story. Eur. J. Med. Res. 15, 571–576 10.1186/2047-783X-15-12-571 21163732 PMC3352106

[B176] Yang C. Zhang W. Liu R. Li Q. Li B. Wang S. (2011). Phylogenetic diversity and metabolic potential of activated sludge microbial communities in full-scale wastewater treatment plants. Environ. Sci. Technol. 45, 7408–7415 10.1021/es2010545 21780771

[B177] Ye L. Zhang T. (2011). Pathogenic bacteria in sewage treatment plants as revealed by 454 pyrosequencing. Environ. Sci. Technol. 45, 7173–7179 10.1021/es201045e 21780772

[B178] Ye L. Zhang T. (2013). Bacterial communities in different sections of a municipal wastewater treatment plant revealed by 16S rDNA 454 pyrosequencing. Appl. Microbiol. Biotechnol. 97, 2681–2690 10.1007/s00253-012-4082-4 22555912 PMC3586070

[B179] Yoon J. W. Lee K. J. Lee S. Y. Chae M. J. Park J. K. Yoo J. H. (2010). Antibiotic resistance profiles of *Staphylococcus pseudintermedius* isolates from Canine patients in Korea. J. Microbiol. Biotechnol. 20, 1764–1768 21193835

[B180] Zhang T. Zhang X.-X. Ye L. (2011). Plasmid metagenome reveals high levels of antibiotic resistance genes and mobile genetic elements in activated sludge. PLoS ONE 6:e26041 10.1371/journal.pone.0026041 22016806 PMC3189950

[B181] Zhang A. Y. Wang H. N. Tian G. B. Zhang Y. Yang X. Xia Q. Q. (2009). Phenotypic and genotypic characterisation of antimicrobial resistance in faecal bacteria from 30 Giant pandas. Int. J. Antimicrob. Agents 33, 456–460 10.1016/j.ijantimicag.2008.10.030 19168331

